# Supplementary material for: Evolutionary games on isothermal graphs
Source: Nat Commun. 2019 Nov 8;10:5107. doi: 10.1038/s41467-019-13006-7 (PMC6841731; doi:10.1038/s41467-019-13006-7)
Supplement: Supplementary file 1 — Supplementary Information [file 41467_2019_13006_MOESM1_ESM.pdf]

Supplementary Information for **"Evolutionary games on isothermal graphs"**

Benjamin Allen, Gabor Lippner, and Martin A. Nowak

Contents

Supplementary Note 1: Derivation of conditions for success **2**
1.1 Model and notation 2
1.1.1 Isothermal graphs 2
1.1.2 Evolutionary process 3
1.2 Weak selection analysis 4
1.3 Coalescence times 5
1.4 Conditions for success: death-Birth or birth-Death 7
1.5 Conditions for success: Birth-death or Death-birth 8
1.6 Extension to arbitrary 2×2 games 8

Supplementary Note 2: Bounds on effective degree **9**
2.1 Background on hitting times 9
2.2 Spectral gap bounds on remeeting times 10
2.3 Quantile bounds for an arbitrary isothermal graph 11
2.4 Isothermal expander graphs 12
2.5 Arithmetic and harmonic mean bounds 13
2.6 Sharper bounds for large spectral gap 14

Supplementary Note 3: Examples **16**
3.1 Wheel graph 16
3.2 Island model 18
3.3 Power-law graphs 21
3.3.1 Quantile bounds 21

3.3.2 Sharper bounds for $g > 1/2$ 22

Supplementary Note 4: Diffusible public goods **23**

Supplementary Note 5: Non-isothermalizable topologies **27**

Supplementary Note 1: Derivation of conditions for success

The conditions for a type to be favored on an arbitrary weighted graph, for weak selection, were derived in Refs. [1, 2]. Here we provide a simplified derivation for the case of isothermal graphs.

1.1 Model and notation

1.1.1 Isothermal graphs

In our model, population structure is described by a weighted isothermal graph G with weights w_{ij}. The graph is undirected ($w_{ij} = w_{ji}$ for each i, j) and has no self-loops ($w_{ii} = 0$ for all i). For all update rules we will consider, transition probabilities depend on relative, rather than absolute, edge weights. Because of this we may scale edge weights so that $\sum_{j \in G} w_{ij} = 1$ for each vertex i, without loss of generality.

The Simpson degree of vertex i is defined as

$$\kappa_i = \left(\sum_{j \in G} w_{ij}^2 \right)^{-1}.$$

Random walks on G are defined with step probabilities equal to edge weights: $p_{ij} = w_{ij}$ for each pair of vertices i and j. The probability that an n-step random walk from i terminates at j is denoted $p_{ij}^{(n)}$. Note that

$$p_{ii}^{(2)} = \sum_{j \in G} w_{ij}^2 = \kappa_i^{-1}.$$

The stationary distribution for random walks on isothermal graphs is uniform: $\pi_i = 1/N$ for each $i \in G$. We also have the reversibility property $p_{ij}^{(n)} = p_{ji}^{(n)}$.

The adjacency matrix of an isothermal graph G is symmetric and doubly stochastic. It therefore has real eigenvalues $1 = \lambda_1 \geq \lambda_2 \geq \ldots \geq \lambda_N$. Moreover, $\lambda_2 < 1$ as long as G is connected. The *spectral gap* is defined as $g = 1 - \lambda_2$.

1.1.2 Evolutionary process

The type occupying vertex i is denoted $x_i \in \{0, 1\}$, with 1 corresponding to A and 0 corresponding to B. The population state is given by the vector $\mathbf{x} = (x_i)_{i \in G} \in \{0, 1\}^G$.

There are two competing types, A and B, corresponding to two strategies in the matrix game:

$$\begin{array}{c} \\ \text{A} \\ \text{B} \end{array} \begin{array}{c} \begin{array}{cc} \text{A} & \text{B} \end{array} \\ \begin{pmatrix} a & b \\ c & d \end{pmatrix} \end{array}, \tag{1}$$

In a given state, the edge-weighted average payoff to vertex i is denoted $f_i(\mathbf{x}) = \sum_{j \in G} w_{ij} f_{ij}(\mathbf{x})$, where $f_{ij}(\mathbf{x})$ is the game payoff that i receives from interacting with j in state $\mathbf{x}$.

Payoff is translated into fecundity by $F_i = 1 + \delta f_i(\mathbf{x})$, where $\delta \geq 0$ quantifies the strength of selection. The case $\delta = 0$ represents neutral drift, for which the game has no effect on selection. Weak selection is the regime $0 < \delta \ll 1$.

Each time-step, an individual in a particular vertex i is chosen to reproduce, and the offspring replaces the occupant of another vertex j. The probability that i replaces j in a given state $\mathbf{x}$, which we denote by $e_{ij}(\mathbf{x})$, depends on the specified update rule:

$$e_{ij}(\mathbf{x}) = \begin{cases} \dfrac{1}{N} \left(\dfrac{w_{ij} F_i(\mathbf{x})}{\sum_{k \in G} w_{kj} F_k(\mathbf{x})} \right) & \text{death-Birth (dB)} \\[2ex] \left(\dfrac{(F_j(\mathbf{x}))^{-1}}{\sum_{k \in G} (F_k(\mathbf{x}))^{-1}} \right) w_{ij} & \text{Death-birth (Db)} \\[2ex] \left(\dfrac{F_i(\mathbf{x})}{\sum_{k \in G} F_k(\mathbf{x})} \right) w_{ij} & \text{Birth-death (Bd)} \\[2ex] \dfrac{1}{N} \left(\dfrac{w_{ij} (F_j(\mathbf{x}))^{-1}}{\sum_{k \in G} w_{ik} (F_k(\mathbf{x}))^{-1}} \right) & \text{birth-Death (bD)} \end{cases} \tag{2}$$

(The above replacement probabilities are stated for isothermal graphs with $\sum_j w_{ij} = 1$ for each i. For arbitrary weighted graphs, the w_{ij} in the above formulae are replaced by $p_{ij} = w_{ij}/\sum_k w_{ik}$. Note that these p_{ij}, and by extension the transition probabilities, are invariant to rescaling all edge weights by a constant.)

For neutral drift ($\delta = 0$), the probability that i replaces j is $e_{ij} = w_{ij}/N$ for all four update rules. This property is particular to isothermal graphs, and does not hold for the more general class of weighted undirected graphs.

There are two absorbing states: the state $\mathbf{1}$ for which only type A is present ($x_i = 1\ \forall i \in G$), and the state $\mathbf{0}$ for which only type B is present ($x_i = 0\ \forall i \in G$). All other states are transient [3, Theorem 2]. We define the *fixation probability of A*, denoted ρ_A, as the expected probability of reaching state $\mathbf{1}$ from an initial state with a single A at a uniformly chosen random vertex, and all other vertices having type B. Likewise, we define the *fixation probability of B*, denoted ρ_B, as the expected probability of reaching state $\mathbf{1}$ from an initial state with a single B at a uniformly chosen random vertex, and all other vertices having type A.

1.2 Weak selection analysis

The expected change in the number of A's from a given state $\mathbf{x}$ can be expressed as

$$\Delta(\mathbf{x}) = \sum_{i,j \in G} e_{ij}(\mathbf{x})(x_i - x_j). \tag{3}$$

We compute this for the four update rules, based on Supplementary Equation 2

$$\Delta(\mathbf{x}) = \begin{cases} \dfrac{1}{N} \displaystyle\sum_{j \in G} \left(-x_j + \frac{\sum_{i \in G} x_i w_{ij} F_i(\mathbf{x})}{\sum_{k \in G} w_{kj} F_k(\mathbf{x})} \right) & \text{dB} \\[2ex] \dfrac{\sum_{j \in G} \left((F_j(\mathbf{x}))^{-1} \left(-x_j + \sum_{i \in G} x_i w_{ij} \right) \right)}{\sum_{k \in G} (F_k(\mathbf{x}))^{-1}} & \text{Db} \\[2ex] \dfrac{\sum_{i \in G} \left(F_i(\mathbf{x}) \left(x_i - \sum_{j \in G} x_j w_{ij} \right) \right)}{\sum_{k \in G} F_k(\mathbf{x})} & \text{Bd} \\[2ex] \dfrac{1}{N} \displaystyle\sum_{i \in G} \left(x_i - \frac{\sum_{j \in G} x_j w_{ij} (F_j(\mathbf{x}))^{-1}}{\sum_{k \in G} w_{ik} (F_k(\mathbf{x}))^{-1}} \right) & \text{bD.} \end{cases} \tag{4}$$

We observe that for neutral drift ($\delta = 0$), $\Delta(\mathbf{x}) = 0$ for each state $\mathbf{x}$. For weak selection, we require the derivative, $\Delta'(\mathbf{x}) = \frac{d\Delta(\mathbf{x})}{d\delta}|_{\delta=0}$. These derivatives can be expressed as

$$\Delta'(\mathbf{x}) = \begin{cases} \frac{1}{N}\sum_{i\in G} x_i \left(f_i(\mathbf{x}) - f_i^{(2)}(\mathbf{x})\right) & \text{dB or bD} \\ \frac{1}{N}\sum_{i\in G} x_i \left(f_i(\mathbf{x}) - f_i^{(1)}(\mathbf{x})\right) & \text{Bd or Db.} \end{cases} \tag{5}$$

Above, we have introduced the notation $f_i^{(n)}(\mathbf{x}) = \sum_j p_{ij}^{(n)} f_j(\mathbf{x})$ for the expected payoff to the vertex at the end of an n-step random walk from i.

We say that A is favored under weak selection if $\rho_A > \rho_B$ to first order in δ. Allen and McAvoy [2] showed this criterion can be evaluated by computing the expectation of $\Delta(\mathbf{x})$ over a particular probability distribution of population states $\mathbf{x}$. This distribution, called the *neutral rare-mutation conditional distribution*, is obtained by (i) fixing $\delta = 0$, (ii) introducing a mutation probability $u > 0$ to obtain a stationary distribution over states, (iii) conditioning this stationary distribution on both types being present, and (iv) taking the $u \to 0$ limit. Denoting expectations over the neutral rare-mutation conditional distribution by $\langle\ \rangle$, Allen and McAvoy [2] showed that A is favored under weak selection if and only if

$$\langle \Delta' \rangle > 0. \tag{6}$$

1.3 Coalescence times

The conditions for success under weak selection can be expressed in terms of *coalescence times*. Coalescence times are defined by considering a discrete-time process in which two random walkers start at vertices i and j. At each time, one of them is chosen, with equal probability, to take a step according to the usual step probabilities p_{ij}. We denote the positions of the walkers at time t by the pair (X_t, Y_t), where X_t and Y_t are random variables with values in G.

Suppose that the two walkers start at vertices i and j: $(X_0, Y_0) = (i, j)$. Let M_{ij} denote the time until the walkers meet: $M_{ij} = \min\{t \geq 0 : X(t) = Y(t)\}$. The *coalescence time* is defined as $\tau_{ij} = \mathbb{E}[M_{ij}]$. These coalescence times satisfy the recurrence relation

$$\tau_{ij} = \begin{cases} 0 & i = j \\ 1 + \frac{1}{2}\sum_{k\in G}\left(w_{ik}\tau_{jk} + w_{jk}\tau_{ik}\right) & i \neq j. \end{cases} \tag{7}$$

For isothermal graphs, this coalescing random walk applies to all four update rules. (For arbitrary weighted graphs, the coalescing random walk varies for different update rules [2, 1].)

Of particular interest is the *remeeting time* for two walkers from the same vertex. Suppose both walkers start at vertex i: $(X_0, Y_0) = (i, i)$. Let M_{ii}^+ denote the first *positive* time for which the walkers occupy the same vertex: $M_{ii}^+ = \min\{t > 0 : X(t) = Y(t)\}$. We define the *remeeting time* as $\tau_i = \mathbb{E}[M_{ii}^+]$. Remeeting times are related to coalescence times by

$$\tau_i = 1 + \sum_{j \in G} w_{ij} \tau_{ij}. \tag{8}$$

Remeeting times satisfy a return-time identity [1], which in the isothermal case is

$$\sum_{i \in G} \tau_i = N^2. \tag{9}$$

Another important quantity is the remeeting time $\tau^{(n)}$ from the two ends of an n-step random walk started from stationarity:

$$\tau^{(n)} = \frac{1}{N} \sum_{i,j \in G} p_{ij}^{(n)} \tau_{ij}. \tag{10}$$

The $\tau^{(n)}$ satisfy the recurrence relation

$$\tau^{(n+1)} = \tau^{(n)} + \frac{1}{N} \sum_{i \in G} p_{ii}^{(n)} \tau_i - 1. \tag{11}$$

Using Supplementary Equations 9 and 11, and recalling the absence of self-loops ($p_{ii}^{(1)} = 0$), we obtain

$$\tau^{(0)} = 0 \tag{12}$$

$$\tau^{(1)} = N - 1 \tag{13}$$

$$\tau^{(2)} = N - 2 \tag{14}$$

$$\tau^{(3)} = N + \frac{1}{N} \sum_{i \in G} \tau_i p_{ii}^{(2)} - 3. \tag{15}$$

Defining the effective degree $\tilde{\kappa}$ as

$$\tilde{\kappa} = \frac{\sum_{i \in G} \tau_i}{\sum_{i \in G} \tau_i \kappa_i^{-1}} = N^2 \left(\sum_i \tau_i \kappa_i^{-1} \right)^{-1} = N^2 \left(\sum_i \tau_i p_{ii}^{(2)} \right)^{-1},$$

we can rewrite Supplementary Equation 15 as

$$\tau^{(3)} = N + N/\tilde{\kappa} - 3. \tag{16}$$

Statistics of spatial assortment can be obtained from coalescence times [4, 5]. Eq. (111) of Allen and McAvoy [2] implies that, for each $i, j \in G$,

$$\tau_{ij} \ \propto \ \frac{1}{2} - \langle x_i x_j \rangle. \tag{17}$$

Let us define $x_i^{(n)} = \sum_{j \in G} p_{ij}^{(n)} x_j$ to be the expected value of the type at the end of an n-step random walk from i. Then from Supplementary Equations 10 and 17 we have

$$\sum_{i \in G} \left\langle x_i \left(x_i^{(m)} - x_i^{(n)} \right) \right\rangle \propto \tau^{(n)} - \tau^{(m)}. \tag{18}$$

1.4 Conditions for success: death-Birth or birth-Death

We will first consider the donation game

$$\begin{array}{c} \\ \text{A} \\ \text{B} \end{array} \!\!\begin{array}{c} \begin{array}{cc} \text{A} & \text{B} \end{array} \\ \begin{pmatrix} b-c & -c \\ b & 0 \end{pmatrix} \end{array}, \tag{19}$$

in which type A pays a cost c to give a benefit b to its partner. We will extend our results to the general game, Supplementary Equation 1, in Supplementary Note 1.6. For the donation game (Supplementary Equation 19), the payoff to vertex i can be written

$$f_i = -c x_i + b x_i^{(1)}. \tag{20}$$

To determine the condition for A to be favored for dB or bD updating, we compute

$$\begin{aligned} \langle \Delta' \rangle &= \frac{1}{N} \sum_{i \in G} \left\langle x_i \left(f_i - f_i^{(2)} \right) \right\rangle \\ &= \frac{1}{N} \sum_{i \in G} \left(-c \left\langle x_i \left(x_i - x_i^{(2)} \right) \right\rangle + b \left\langle x_i \left(x_i^{(1)} - x_i^{(3)} \right) \right\rangle \right). \end{aligned}$$

Applying Supplementary Equations 6 and 18, we obtain the condition

$$-c\tau^{(2)} + b\left(\tau^{(3)} - \tau^{(1)}\right) > 0. \tag{21}$$

Now using Supplementary Equations 13–15, we find that A is favored under weak selection if and only if

$$-c(N-2) + b\left(N/\tilde{\kappa} - 2\right) > 0. \tag{22}$$

1.5 Conditions for success: Birth-death or Death-birth

For Bd or Db updating, we compute

$$\begin{aligned}\langle \Delta' \rangle &= \frac{1}{N}\sum_{i\in G}\left\langle x_i\left(f_i - f_i^{(1)}\right)\right\rangle \\ &= \frac{1}{N}\sum_{i\in G}\left(-c\left\langle x_i\left(x_i - x_i^{(1)}\right)\right\rangle + b\left\langle x_i\left(x_i^{(1)} - x_i^{(2)}\right)\right\rangle\right).\end{aligned}$$

Applying Supplementary Equations 6 and 18 yields the condition

$$-c\tau^{(1)} + b\left(\tau^{(2)} - \tau^{(1)}\right) > 0. \tag{23}$$

Substituting from Supplementary Equations 13–15, we obtain that A is favored under weak selection if and only if

$$-c(N-1) - b > 0. \tag{24}$$

1.6 Extension to arbitrary 2×2 games

We turn now to the general 2×2 game, given in Supplementary Equation 1. The Structure Coefficient Theorem [6] states that, for a general class of evolutionary game theory processes, including those considered here, the condition for success under weak selection takes the form

$$\sigma a + b > c + \sigma d, \tag{25}$$

for some structure coefficient σ that is independent of the game matrix. Defining $C = -\frac{1}{2}(a+b-c-d)$ and $B = \frac{1}{2}(a-b+c-d)$, we observe that Supplementary Equation 25 becomes equivalent to

$$-(\sigma+1)C + (\sigma-1)B > 0. \tag{26}$$

The value of σ for a given graph and update rule can be determined by comparing Supplementary Equation 25 to Supplementary Equations 22 and 24 in the case of the donation game (Supplementary Equation 19). Solving for σ yields

$$\sigma = \begin{cases} \frac{\tilde{\kappa}+1-4\tilde{\kappa}/N}{\tilde{\kappa}-1} & \text{dB or bD} \\ (N-2)/N & \text{Bd or Db.} \end{cases} \tag{27}$$

Combining Supplementary Equations 25 and 27 gives Eqs. (4) and (9) of the main text. Combining Supplementary Equations 26 and 27 and simplifying gives the conditions

$$\begin{cases} -(N-1)C - B > 0 & \text{Bd or Db} \\ -C(N-2) + B\left(N/\tilde{\kappa} - 2\right) > 0 & \text{dB or bD,} \end{cases} \tag{28}$$

as stated in Eqs. (5) and (10) of the main text.

Supplementary Note 2: Bounds on effective degree

Here we derive upper and lower bounds on the remeeting time τ_i from a single vertex, in terms of the spectral gap g, and use these to obtain bounds on the effective degree $\tilde{\kappa}$.

2.1 Background on hitting times

For a single random walk on G, let h_{ij} denote the expected hitting time to vertex j when starting from vertex i. These hitting times satisfy the recurrence equations

$$\begin{cases} h_{ij} = 1 + \sum_k w_{ik} h_{kj} & \text{for } i \neq j, \\ h_{ii} = 0 & \text{for all } i. \end{cases} \tag{29}$$

Since the stationary distribution on isothermal graphs is uniform, it follows from the return-time identity (e.g. [7, Lemma 2.5]) that the expected time for a random walk to return to its initial vertex is N, regardless of which initial vertex is used. Combining with Supplementary Equation 29, we have the identity

$$1 + \sum_k w_{ik} h_{ik} = N. \tag{30}$$

Let $h_{*j} = \frac{1}{N}\sum_i h_{ij}$ denote the expected hitting time to j from a vertex chosen uniformly at random. Corollary 3.14 of Ref. [7] gives the identity

$$h_{ij} - h_{*j} = h_{ji} - h_{*i}. \tag{31}$$

Proposition 3.17 of Ref. [7] gives bounds on h_{*j} in terms of the spectral gap. In the case of an isothermal graph with no self-loops, these bounds are

$$\frac{(N-1)^2}{N} \leq h_{*j} \leq \frac{N-1}{g}, \tag{32}$$

for all vertices j.

2.2 Spectral gap bounds on remeeting times

We ultilize the bounds in Supplementary Equation 32 to obtain bounds on the remeeting times τ_i.

Theorem. *For each $i \in G$,*

$$2N - \frac{N-1}{g} - \frac{2N-1}{N} \leq \tau_i \leq \frac{N-1}{g} + \frac{2N-1}{N}. \tag{33}$$

We note that the lower bound in Supplementary Equation 33 is not necessarily positive.

Proof. Our proof is a variation on the proof of Proposition 14.5 of Ref. [7]. Consider the process of two random walkers $(X_t, Y_t)_{t=0}^{\infty}$ described in Supplementary Note 1.3, where both walkers start at vertex i: $(X_0, Y_0) = (i, i)$. We define the real-valued stochastic process $(S_t)_{t=0}^{\infty}$ by

$$S_t = \begin{cases} N - h_{*i} & t = 0 \\ t + h_{X_t Y_t} - h_{*Y_t} & t \geq 1 \end{cases} \tag{34}$$

Supplementary Equations 29, 30, and 31 imply that S_t for $0 \leq t \leq M_{ii}^+$ is a Martingale. We then have

$$\begin{aligned} N - h_{*i} &= S_0 \\ &= \mathbb{E}[S_{M_{ii}^+}] && \text{by the Optional Stopping Theorem [8]} \\ &= \mathbb{E}[M_{ii}^+] - \mathbb{E}\left[h_{*Y_{M_{ii}^+}}\right] && \text{by the construction of } S_t \\ &= \tau_i - \mathbb{E}\left[h_{*Y_{M_{ii}^+}}\right] && \text{by definition of } \tau_i. \end{aligned}$$

Rearranging,

$$\tau_i = N - h_{*i} + \mathbb{E}\left[h_{*Y_{M_{ii}^+}}\right]. \tag{35}$$

The upper bound on τ_i is obtained by applying the lower bound in Supplementary Equation 32 to the second term of Supplementary Equation 35, and the upper bound in Supplementary Equation 32 to the third term of Supplementary Equation 35. Applying these bounds in the opposite fashion gives the lower bound on τ_i. □

We note that the average of the upper and lower bounds in Supplementary Equation 33 is N, which is equal to the average value of τ_i according to Supplementary Equation 9. We also observe that as $N \to \infty$, the lower and upper bounds are asymptotically $N(2-g^{-1})+\mathcal{O}(1)$ and $Ng^{-1}+\mathcal{O}(1)$, respectively. Thus, in the $N \to \infty$ limit, the lower bound is relevant (i.e., positive) for $g > 1/2$.

2.3 Quantile bounds for an arbitrary isothermal graph

Recall that $\tilde{\kappa}$ is a weighted harmonic average of the Simpson degrees κ_i. The weights τ_i are bounded by Supplementary Equation 33. Upper and lower bounds on $\tilde{\kappa}$ can therefore be obtained by placing the maximum weight on vertices that have the largest or smallest Simpson degrees, respectively, taking into account that the sum of the τ_i is constrained by Supplementary Equation 9.

To formalize this idea, we introduce the quantity

$$\hat{g} = \left(\frac{N-1}{Ng} + \frac{2N-1}{N^2}\right)^{-1}, \tag{36}$$

so that the bounds in Supplementary Equation 33 become

$$N(2-\hat{g}^{-1}) \leq \tau_i \leq N\hat{g}^{-1}. \tag{37}$$

We note that $\hat{g} = g + \mathcal{O}(N^{-1})$ as $N \to \infty$, and also that $\hat{g} > g$ as long as $g < 1/2$.

We write the effective degree of G in the form

$$\tilde{\kappa} = \left(\sum_{i \in G}\left(\frac{\tau_i}{N^2}\right)\kappa_i^{-1}\right)^{-1}. \tag{38}$$

By Supplementary Equations 9 and 33, the weights, τ_i/N^2, are subject to the constraints

$$\frac{\tau_i}{N^2} \leq \frac{1}{N\hat{g}}, \qquad \sum_{i \in G} \frac{\tau_i}{N^2} = 1. \tag{39}$$

Next we index the vertices in order of increasing Simpson degree, so that

$$\kappa_1 \leq \kappa_2 \leq \cdots \leq \kappa_N. \tag{40}$$

We define $\mathrm{H}_{[0,\hat{g}]}[\kappa]$ to be the harmonic average over the fraction $\hat{g}$ of vertices with the smallest Simpson degree:

$$\mathrm{H}_{[0,\hat{g}]}[\kappa] = \left(\frac{1}{N\hat{g}} \sum_{i=1}^{\lfloor N\hat{g} \rfloor} \kappa_i^{-1} + \left(1 - \frac{\lfloor N\hat{g} \rfloor}{N\hat{g}}\right) \kappa_{\lfloor N\hat{g} \rfloor + 1}^{-1} \right)^{-1}. \tag{41}$$

Above, $\lfloor N\hat{g} \rfloor$ denotes the greatest integer less than or equal to $N\hat{g}$ (i.e. the floor function of $N\hat{g}$). In Supplementary Equation 41, the $\lfloor N\hat{g} \rfloor$ vertices with the smallest Simpson degree are each given weight $1/(N\hat{g})$. The remainder of the weight is placed on the vertex with next-smallest Simpson degree $\kappa_{\lfloor N\hat{g} \rfloor + 1}$, so that the total weights sum to one.

Similarly, we define $\mathrm{H}_{[1-\hat{g},1]}[\kappa]$ to be the harmonic average over the fraction $\hat{g}$ of vertices with the largest Simpson degree:

$$\mathrm{H}_{[1-\hat{g},1]}[\kappa] = \left(\left(1 - \frac{\lfloor N\hat{g} \rfloor}{N\hat{g}}\right) \kappa_{N - \lfloor N\hat{g} \rfloor}^{-1} + \frac{1}{N\hat{g}} \sum_{i=N-\lfloor N\hat{g} \rfloor + 1}^{N} \kappa_i^{-1} \right)^{-1}. \tag{42}$$

$\mathrm{H}_{[0,\hat{g}]}[\kappa]$ and $\mathrm{H}_{[1-\hat{g},1]}[\kappa]$ represent the smallest and largest values, respectively, of the right-hand side of Supplementary Equation 38 that are achievable under the constraints of Supplementary Equation 39. We therefore have

$$\mathrm{H}_{[0,\hat{g}]}[\kappa] \leq \tilde{\kappa} \leq \mathrm{H}_{[1-\hat{g},1]}[\kappa]. \tag{43}$$

2.4 Isothermal expander graphs

We now consider a sequence of isothermal graphs $\{G_j\}_{j=1}^{\infty}$ with corresponding sizes N_j and spectral gaps g_j. We define this to be a sequence of isothermal *expander* graphs if $\lim_{j\to\infty} N_j = \infty$ and $\liminf_{j\to\infty} g_j > 0$. By passing to a subsequence if necessary, we can assume $\lim_{j\to\infty} g_j = g > 0$. Notice that this also entails $\lim_{j\to\infty} \hat{g}_j = g$.

To obtain limiting values of the bounds in Supplementary Equation 43 for such a sequence, we turn the degree sequence for graph G_j (Supplementary Equation 40) into a quantile function—a nondecreasing piecewise-constant function $\kappa_j(x)$, defined for $0 < x \leq 1$. Let $\kappa_{i,j}$ denote the ith smallest Simpson degree among the vertices of G_j. We then define $\kappa_j(x) = \kappa_{\lceil N_j x \rceil, j}$, where $\lceil\ \rceil$ denotes the ceiling function. Supplementary Equations 41 and 42 can then be rewritten as

$$\mathrm{H}_{[0,\hat{g}_j]}[\kappa_j] = \left(\hat{g}_j^{-1} \int_0^{\hat{g}_j} \big(\kappa_j(x)\big)^{-1}\, dx \right)^{-1} \tag{44}$$

$$\mathrm{H}_{[1-\hat{g}_j,1]}[\kappa_j] = \left(\hat{g}_j^{-1} \int_{1-\hat{g}_j}^{1} \big(\kappa_j(x)\big)^{-1}\, dx \right)^{-1}. \tag{45}$$

Now suppose that as $j \to \infty$, $\kappa_j(x)$ converges pointwise to some real-valued function $\kappa(x)$ on the interval $[0,1)$. Then $\kappa(x)$ is the quantile function of the limiting Simpson degree distribution. That is, a fraction x of the Simpson degrees lie below $\kappa(x)$, for each $0 \leq x < 1$. We allow for the possibility that $\lim_{x\to 1} \kappa(x) = \infty$.

As $j \to \infty$, the bounds in Supplementary Equation 43 converge to

$$\kappa_{[0,g]} \leq \tilde{\kappa} \leq \kappa_{[1-g,1]}, \tag{46}$$

with the limiting bounds given by the limits of Supplementary Equations 44 and 45, respectively:

$$\kappa_{[0,g]} = \lim_{j\to\infty} \mathrm{H}_{[0,\hat{g}_j]}[\kappa_j] = \left(g^{-1} \int_0^{g} \big(\kappa(x)\big)^{-1}\, dx \right)^{-1} \tag{47}$$

$$\kappa_{[1-g,1]} = \lim_{j\to\infty} \mathrm{H}_{[1-\hat{g}_j,1]}[\kappa_j] = \left(g^{-1} \int_{1-g}^{1} \big(\kappa(x)\big)^{-1}\, dx \right)^{-1}. \tag{48}$$

Convergence is guaranteed by the Dominated Convergence Theorem, using the fact that $0 < \big(\kappa_j(x)\big)^{-1} \leq 1$ for all $j \geq 1$ and all $x \in [0,1)$. Supplementary Equation 46 is Eq. (6) of the main text.

2.5 Arithmetic and harmonic mean bounds

We can also obtain simpler, but looser, bounds that depend only on the arithmetic and harmonic mean Simpson degree. We begin with a single

isothermal graph G. For a lower bound, using Supplementary Equation 37, we have

$$\tilde{\kappa} = \left(\sum_{i \in G} \frac{\tau_i}{N^2} \kappa_i^{-1} \right)^{-1} \geq \left(\frac{1}{N\hat{g}} \sum_{i \in G} \kappa_i^{-1} \right)^{-1} = \hat{g}\bar{\kappa}_{\mathrm{H}}, \tag{49}$$

where $\bar{\kappa}_{\mathrm{H}}$ denotes the (unweighted) harmonic average Simpson degree.

For the upper bound, we have the following series of inequalities:

$$\mathrm{H}_{[1-\hat{g},1]}[\kappa] \leq \mathrm{A}_{[1-\hat{g},1]}[\kappa] \leq \frac{1}{\hat{g}} \bar{\kappa}_{\mathrm{A}}. \tag{50}$$

Above, $\mathrm{A}_{[1-\hat{g},1]}[\kappa]$ is the arithmetic average of the fraction $\hat{g}$ of Simpson degrees that are the largest, defined similarly to $\mathrm{H}_{[1-\hat{g},1]}[\kappa]$. $\bar{\kappa}_{\mathrm{A}} = \mathrm{A}_{[0,1]}[\kappa]$ is the average Simpson degree over all vertices. The first inequality in Supplementary Equation 50 is the arithmetic-harmonic means inequality, while the second reflects the fact that $\frac{1}{\hat{g}}\bar{\kappa}_{\mathrm{A}}$ can be obtained by adding additional positive terms to $\mathrm{A}_{[1-\hat{g},1]}[\kappa]$.

Combining Supplementary Equations 46, 49, and 50, we have

$$\hat{g}\bar{\kappa}_{\mathrm{H}} \leq \tilde{\kappa} \leq \frac{\bar{\kappa}_{\mathrm{A}}}{\hat{g}}. \tag{51}$$

Turning now to a sequence of isothermal expander graphs $\{G_j\}_{j=1}^{\infty}$, the bounds in Supplementary Equation 51 converge to

$$g\bar{\kappa}_{\mathrm{H}} \leq \tilde{\kappa} \leq \frac{\bar{\kappa}_{\mathrm{A}}}{g}. \tag{52}$$

This is Eq. (7) of the main text. We note that, for any given graph with spectral gap $g < 1/2$, the bounds in Supplementary Equation 51 are stronger than those in Supplementary Equation 52, since $\hat{g} > g$ in this case. We also note that $\bar{\kappa}_{\mathrm{A}}$ may diverge as $j \to \infty$; thus the upper bound in Supplementary Equation 52 may be infinity.

2.6 Sharper bounds for large spectral gap

In the case that the lower bound on τ_i in Supplementary Equation 37 is positive, we can further sharpen the bounds on the effective degree by assigning one bound in Supplementary Equation 37 to the lower half of Simpson degrees and the other bound to the upper half. Specifically, suppose the vertices

are ordered as in Supplementary Equation 40. Then a lower bound is given by

$$\tilde{\kappa} \geq \left(\frac{\hat{g}^{-1}}{N} \sum_{i=1}^{N/2} \kappa_i^{-1} + \frac{2-\hat{g}^{-1}}{N} \sum_{i=N/2+1}^{N} \kappa_i^{-1} \right)^{-1}, \tag{53}$$

if N is even, and

$$\tilde{\kappa} \geq \left(\frac{\hat{g}^{-1}}{N} \sum_{i=1}^{(N-1)/2} \kappa_i^{-1} + \frac{\kappa_{(N+1)/2}^{-1}}{N} + \frac{2-\hat{g}^{-1}}{N} \sum_{i=(N+3)/2}^{N} \kappa_i^{-1} \right)^{-1}. \tag{54}$$

if N is odd. Similarly, for an upper bound, we have

$$\tilde{\kappa} \leq \left(\frac{2-\hat{g}^{-1}}{N} \sum_{i=1}^{N/2} \kappa_i^{-1} + \frac{\hat{g}^{-1}}{N} \sum_{i=N/2+1}^{N} \kappa_i^{-1} \right)^{-1}, \tag{55}$$

if N is even, and

$$\tilde{\kappa} \leq \left(\frac{2-\hat{g}^{-1}}{N} \sum_{i=1}^{(N-1)/2} \kappa_i^{-1} + \frac{\kappa_{(N+1)/2}^{-1}}{N} + \frac{\hat{g}^{-1}}{N} \sum_{i=(N+3)/2}^{N} \kappa_i^{-1} \right)^{-1}. \tag{56}$$

if N is odd.

If we now consider a family of isothermal expander graphs $\{G_j\}_{j=1}^{\infty}$ as in the previous section, with limiting spectral gap $g > 0$ and limiting degree sequence described by the real-valued function $\kappa(x)$, the corresponding bounds on the effective degree converge to

$$\left(\frac{1}{2g} \bar{\kappa}_{\text{low}}^{-1} + \left(1 - \frac{1}{2g}\right) \bar{\kappa}_{\text{high}}^{-1} \right)^{-1} \leq \tilde{\kappa} \leq \left(\left(1 - \frac{1}{2g}\right) \bar{\kappa}_{\text{low}}^{-1} + \frac{1}{2g} \bar{\kappa}_{\text{high}}^{-1} \right)^{-1}, \tag{57}$$

where $\bar{\kappa}_{\text{low}} = \mathrm{H}_{\left[0,\frac{1}{2}\right]}[\kappa]$ and $\bar{\kappa}_{\text{high}} = \mathrm{H}_{\left[\frac{1}{2},1\right]}[\kappa]$ are the harmonic averages of the smaller and larger half of Simpson degrees, respectively. The bounds in Supplementary Equation 57 can also be written as

$$\frac{2g\bar{\kappa}_{\text{low}}\bar{\kappa}_{\text{high}}}{(2g-1)\bar{\kappa}_{\text{low}} + \bar{\kappa}_{\text{high}}} \leq \tilde{\kappa} \leq \frac{2g\bar{\kappa}_{\text{low}}\bar{\kappa}_{\text{high}}}{\bar{\kappa}_{\text{low}} + (2g-1)\bar{\kappa}_{\text{high}}}. \tag{58}$$

Supplementary Note 3: Examples

3.1 Wheel graph

The isothermal wheel graph (Fig. 1B of the main text) has n wheel vertices and one hub. Neighboring wheel vertices are joined by edges of weight $(n-1)/(2n)$, and each wheel vertex is joined to the hub by an edge of weight $1/n$. Let $\tau_{L,j}$ denote the coalescence time for two leaves that are j apart, $0 \leq j \leq n$. Clearly, $\tau_{L,0} = \tau_{L,n} = 0$. Let τ_{LH} denote the coalescence time between a leaf and the hub.

The recurrence relation for coalescence times (Supplementary Equation 7) becomes

$$\tau_{L,j} = 1 + \frac{n-1}{2n}\left(\tau_{L,j-1} + \tau_{L,j+1}\right) + \frac{1}{n}\tau_{LH} \quad \text{for } 1 \leq j \leq n-1, \tag{59}$$

$$\tau_{LH} = 1 + \frac{n-1}{2n}\tau_{LH} + \frac{1}{2n}\sum_{j=0}^{n-1}\tau_{L,j}. \tag{60}$$

For convenience, we define $\tau'_{L,j} = \tau_{L,j} - \tau_{LH}$. Then Supplementary Equations 59–60 become

$$\tau'_{L,j} = 1 + \frac{n-1}{2n}\left(\tau'_{L,j-1} + \tau'_{L,j+1}\right) \quad \text{for } 1 \leq j \leq n-1, \tag{61}$$

$$\tau_{LH} = 2n + \sum_{j=0}^{n-1}\tau'_{L,j}. \tag{62}$$

As an *ansatz*, we suppose the solution to Supplementary Equation 61 takes the form

$$\tau'_{L,j} = a + b\left(\gamma^j + \gamma^{n-j}\right), \tag{63}$$

for some a, b, γ depending on n but not on j. Substituting into Supplementary Equation 61 gives

$$\begin{aligned} a + b\left(\gamma^j + \gamma^{n-j}\right) &= 1 + \frac{n-1}{2n}\left(2a + b\left(\gamma^{j-1} + \gamma^{n-j+1} + \gamma^{j+1} + \gamma^{n-j-1}\right)\right) \\ &= 1 + \frac{n-1}{2n}\left(2a + b\left(\gamma + \gamma^{-1}\right)\left(\gamma^j + \gamma^{n-j}\right)\right). \end{aligned}$$

For this to hold for all $1 \leq j \leq n-1$ necessitates that

$$a = 1 + \frac{n-1}{n}a \quad \text{and} \quad (n-1)\left(\gamma + \gamma^{-1}\right) = 2n.$$

Solving the above equations yields $a = n$ and

$$\gamma = \frac{n - \sqrt{2n-1}}{n-1}, \qquad \gamma^{-1} = \frac{n + \sqrt{2n-1}}{n-1}.$$

To solve for b, we substitute into Supplementary Equation 62,

$$\begin{aligned} \tau_{LH} &= 2n + \sum_{j=0}^{n-1} \left(n + b\left(\gamma^j + \gamma^{n-j}\right)\right) \\ &= n(n+2) + b\frac{(1+\gamma)(1-\gamma^n)}{1-\gamma}. \end{aligned} \tag{64}$$

Additionally, since $\tau_{L,0} = 0$, we have

$$\tau_{LH} = -\tau'_{L,0} = -n - b(1+\gamma^n). \tag{65}$$

Combining Supplementary Equations 64 and 65 and solving for b yields

$$b = -\frac{n(n+3)}{2}\left(\frac{1-\gamma}{1-\gamma^{n+1}}\right).$$

Substituting this value of b into Supplementary Equations 65 and 63, we obtain the coalescence times

$$\begin{aligned} \tau_{LH} &= \frac{n(n+3)}{2}\left(\frac{(1-\gamma)(1+\gamma^n)}{1-\gamma^{n+1}}\right) - n, \\ \tau_{L,j} &= \tau'_{L,j} + \tau_{LH} \\ &= \frac{n(n+3)}{2}\left(\frac{(1-\gamma)(1-\gamma^j)(1-\gamma^{n-j})}{1-\gamma^{n+1}}\right). \end{aligned}$$

In particular, for neighboring leaves ($j = 1$), we have

$$\tau_{L,1} = \frac{n(n+3)}{2}\left(\frac{(1-\gamma)^2(1-\gamma^{n-1})}{1-\gamma^{n+1}}\right). \tag{66}$$

Turning now to remeeting times, we compute

$$\begin{aligned} \tau_H &= 1 + \tau_{LH} \\ &= \frac{n(n+3)}{2}\left(\frac{(1-\gamma)(1+\gamma^n)}{1-\gamma^{n+1}}\right) - (n-1), \\ \tau_L &= 1 + \tfrac{1}{n}\tau_{LH} + \tfrac{n-1}{n}\tau_{L,1} \\ &= \frac{n+3}{2}\left(\frac{(1-\gamma)(n(1+\gamma^n) - (n-1)(\gamma+\gamma^{n-1}))}{1-\gamma^{n+1}}\right). \end{aligned}$$

The Simpson degrees are

$$\kappa_H = n, \qquad \kappa_L = \left(2\frac{(n-1)^2}{4n^2} + \frac{1}{n^2}\right)^{-1} = \frac{2n^2}{(n-1)^2+2}.$$

Using the above values, the effective degree can be calculated as

$$\tilde{\kappa} = \frac{(n+1)^2}{\tau_H \kappa_H^{-1} + n\tau_L\kappa_L^{-1}}. \tag{67}$$

Asymptotically, as $n \to \infty$, we have

$$\tau_H \sim \frac{n\sqrt{n}}{\sqrt{2}}, \qquad \kappa_H \sim n, \qquad \tau_L \sim n, \qquad \kappa_L \sim 2,$$

where $f(n) \sim g(n)$ means that $\lim_{n\to\infty} f(n)/g(n) = 1$. (The asymptotic expressions for τ_H and τ_L were obtained with the aid of Mathematica.) Substituting into Supplementary Equation 67 and simplifying gives $\lim_{n\to\infty} \tilde{\kappa} = 2$.

3.2 Island model

Here we analyze the island model, shown in Fig. 1C of the main text and discussed in 'Promoters of cooperation with infinite average degree' under main text results. The model begins with n separate isothermal graphs ("islands") $G_1, \ldots, G_n$, of respective sizes $N_1, \ldots, N_n$. Each individual island is vertex-transitive, but the size and graph structure may vary across islands. Since each island is vertex-transitive, all vertices of a given island G_x have the same Simpson degree κ_x (but the κ_x may differ of across islands).

The islands are joined into an overall isothermal graph G by adding an edge of weight $\alpha \ll 1$ between each pair of vertices on different islands. To maintain a weighted degree of one, the edges within each island G_x are rescaled by the factor $1 - \alpha(N - N_x)$.

Since each island is vertex-transitive, and each inter-island pair is equally connected (by weight α), the coalescence time τ_{ij} from different islands G_x and G_y depends only on x and y, and not on the particular vertices $i \in G_x$ and $j \in G_y$. Accordingly, we let $\tau_{G_xG_y}$ denote the meeting time between a vertex of G_x and a vertex of G_y, $y \neq x$.

From Supplementary Equation 7 we have the following recurrence for coalescence times. For a pair $i \neq j$ on a common island G_x, we have

$$\tau_{ij} = 1 + \frac{1}{2}\left(1 - \alpha(N - N_x)\right) \sum_{k \in G_x} (w_{ik}\tau_{jk} + w_{jk}\tau_{ik}) + \sum_{y \neq x} \alpha N_y \tau_{G_xG_y}. \tag{68}$$

For inter-island meeting times, we have that for $x \neq y$,

$$\tau_{G_xG_y} = 1 + \frac{\alpha}{2N_x}\sum_{i,j\in G_y}\tau_{ij} + \frac{\alpha}{2N_y}\sum_{i,j\in G_x}\tau_{ij} + \frac{\alpha}{2}\sum_{z\notin\{x,y\}} N_z\left(\tau_{G_zG_y} + \tau_{G_xG_z}\right) + \left(1 - \frac{(N-N_x)\alpha}{2} - \frac{(N-N_y)\alpha}{2}\right)\tau_{G_xG_y}. \quad (69)$$

We seek an asymptotic solution as $\alpha \to 0$. As an *ansatz*, we suppose

$$\begin{cases} \tau_{ij} = \mathcal{O}(1) & \text{for } i \neq j \text{ and } i,j \in G_x \text{ for some } x, \\ \tau_{G_xG_y} = T_{xy}\alpha^{-1} + \mathcal{O}(1) & \text{for } x \neq y, \end{cases} \quad (70)$$

for some collection of values T_{xy}. Substituting in Supplementary Equation 69 and taking $\alpha \to 0$, we find that the T_{xy} satisfy

$$\frac{2N - N_x - N_y}{2}T_{xy} = 1 + \frac{1}{2}\sum_{z\notin\{x,y\}} N_z(T_{xz} + T_{yz}). \quad (71)$$

Defining $T_{xx} = 0$ for all $x = 1, \ldots, n$, we can rewrite Supplementary Equation 71 as

$$NT_{xy} = 1 + \frac{1}{2}\sum_{z=1}^{n} N_z(T_{xz} + T_{yz}). \quad (72)$$

We have not found a general closed-form solution to Supplementary Equation 72. However, if there are $n = 2$ islands, or if all islands have the same size, N/n, the solution is $T_{xy} = n/N$ for all pairs x, y with $x \neq y$.

Taking $\alpha \to 0$ in Supplementary Equation 68 yields

$$\tau_{ij} = 1 + \sum_{y=1}^{n} N_y T_{xy} + \frac{1}{2}\sum_{k\in G_x}(w_{ik}\tau_{jk} + w_{jk}\tau_{ik}). \quad (73)$$

This is the same as the recurrence (Supplementary Equation 7) for coalescence times on island G_x alone, except the time increment is $1 + \sum_{y\neq x} N_y T_{xy}$ instead of 1. It follows that, when the islands are joined to form the overall graph G, all coalescence times from pairs $i, j \in G_x$ are scaled by the factor $1 + \sum_{y=1}^{n} N_y T_{xy}$. This scaling applies to remeeting times as well, so that if $\hat{\tau}_i$

is the remeeting time from vertex i on graph G_x alone, the remeeting time from i on the overall graph G is

$$\tau_i = \left(1 + \sum_{y=1}^{n} N_y T_{xy}\right) \hat{\tau}_i.$$

We check this solution by computing the overall sum of remeeting times, which should equal N^2 by Supplementary Equation 9:

$$\begin{aligned}\sum_{i \in G} \tau_i &= \sum_{x=1}^{n} \left(1 + \sum_{y=1}^{n} N_y T_{xy}\right) \sum_{i \in G_x} \hat{\tau}_i \\ &= \sum_{x=1}^{n} \left(1 + \sum_{y=1}^{n} N_y T_{xy}\right) N_x^2 \\ &= \sum_{x=1}^{n} N_x^2 + \sum_{x,y} N_x^2 N_y T_{xy}. \end{aligned} \tag{74}$$

To show this is equal to N^2, we multiply Supplementary Equation 72 by $N_x N_y$ and sum over all pairs x, y with $x \neq y$:

$$\begin{aligned} N \sum_{\substack{x,y \\ x \neq y}} N_x N_y T_{xy} &= \sum_{\substack{x,y \\ x \neq y}} N_x N_y + \frac{1}{2} \sum_{\substack{x,y,z \\ x \neq y}} N_x N_y N_z (T_{xz} + T_{yz}) \\ N \sum_{x,y} N_x N_y T_{xy} &= N - \sum_{x=1}^{n} N_x^2 + \frac{1}{2} \sum_{x,y,z} N_x N_y N_z (T_{xz} + T_{yz}) - \sum_{x,z} N_x^2 N_z T_{xz} \\ &= N - \sum_{x=1}^{n} N_x^2 + N \sum_{x,y} N_x N_y T_{xy} - \sum_{x,y} N_x^2 N_y T_{xy}. \end{aligned}$$

This yields the identity

$$\sum_{x,y} N_x^2 N_y T_{xy} = N^2 - \sum_{x=1}^{n} N_x^2. \tag{75}$$

Substituting in Supplementary Equation 74 yields $\sum_{i \in G} \tau_i = N^2$ as desired.

We compute the effective degree $\tilde{\kappa}$ of G as

$$\begin{aligned}\tilde{\kappa} &= N^2 \left(\sum_{x=1}^{n} \sum_{i \in G_x} \tau_i \kappa_i^{-1} \right)^{-1} \\ &= N^2 \left(\sum_{x=1}^{n} \left(1 + \sum_{y=1}^{n} N_y T_{xy} \right) \kappa_x^{-1} \sum_{i \in G_x} \hat{\tau}_i \right)^{-1} \\ &= N^2 \left(\sum_{x=1}^{n} \left(1 + \sum_{y=1}^{n} N_y T_{xy} \right) N_x^2 \kappa_x^{-1} \right)^{-1}.\end{aligned}$$

So the effective degree $\tilde{\kappa}$ is a weighted harmonic average of the Simpson degrees on the separate islands, with each κ_x weighted by $N_x^2 \left(1 + \sum_{y=1}^{n} N_y T_{xy}\right)$.

In the case that all islands have equal size, ($N_x = N/n$ for all x), the islands are weighted equally. For $n = 2$ islands (not necessarily of equal size), substituting the solution $T_{12} = 2/N$ to Supplementary Equation 72, we obtain

$$\begin{aligned}\tilde{\kappa} &= N^2 \left(N_1^2 (1 + 2N_2/N) \kappa_1^{-1} + N_2^2 (1 + 2N_1/N) \kappa_2^{-1} \right) \\ &= N^3 \left(N_1^2 (N_1 + 3N_2) \kappa_1^{-1} + N_2^2 (3N_1 + N_2) \kappa_2^{-1} \right)^{-1}.\end{aligned}$$

3.3 Power-law graphs

We now turn to the family of power-law isothermal expander graphs discussed in 'Promoters of cooperation with infinite average degree' under the main text results. Suppose that, for a family of isothermal expander graphs with limiting spectral gap $g > 0$, the Simpson degree distribution converges to the power-law density $f(\kappa) = (\gamma - 1)\kappa_0^{\gamma-1} \kappa^{-\gamma}$, valid for $\kappa \in [\kappa_0, \infty)$, with exponent $\gamma \geq 2$.

3.3.1 Quantile bounds

To apply the quantile bounds (Supplementary Equation 46) to this family, we must determine the quantile function $\kappa(x)$. For this we solve the equation

$$\begin{aligned}x &= (\gamma - 1)\kappa_0^{\gamma-1} \int_{\kappa_0}^{y} \kappa^{-\gamma} \, d\kappa \\ &= 1 - (y/\kappa_0)^{1-\gamma}.\end{aligned}$$

Solving for y yields the quantile function:

$$\kappa(x) = y = \kappa_0 (1-x)^{1/(1-\gamma)}. \tag{76}$$

Now we calculate

$$\begin{aligned}
\mathrm{H}_{[0,g]}[\kappa] &= \left(g^{-1} \int_{\kappa_0}^{\kappa(g)} f(\kappa)\kappa^{-1} d\kappa \right)^{-1} \\
&= \left(g^{-1}(\gamma-1)\kappa_0^{\gamma-1} \int_{\kappa_0}^{\kappa_0(1-g)^{1/(1-\gamma)}} \kappa^{-\gamma-1}\, d\kappa \right)^{-1} \\
&= \left(\frac{\kappa_0 \gamma}{\gamma-1} \right) \frac{g}{1-(1-g)^{\gamma/(\gamma-1)}}.
\end{aligned} \tag{77}$$

Similarly,

$$\begin{aligned}
\mathrm{H}_{[1-g,1]}[\kappa] &= \left(g^{-1} \int_{\kappa(1-g)}^{\infty} f(\kappa)\kappa^{-1} \right)^{-1} \\
&= \left(g^{-1}(\gamma-1)\kappa_0^{\gamma-1} \int_{\kappa_0 g^{1/(1-\gamma)}}^{\infty} \kappa^{-\gamma-1} \right)^{-1} \\
&= \frac{\kappa_0 \gamma}{\gamma-1} g^{-1/(\gamma-1)}.
\end{aligned} \tag{78}$$

Thus we have the bounds

$$\left(\frac{\gamma}{\gamma-1} \right) \frac{\kappa_0 g}{1-(1-g)^{\gamma/(\gamma-1)}} \leq \left(\frac{b}{c} \right)^* \leq \left(\frac{\gamma}{\gamma-1} \right) \kappa_0 g^{-1/(\gamma-1)}. \tag{79}$$

Note that for all $\gamma > 1$, as $g \to 1$, both bounds approach the harmonic mean Simpson degree, which is $\bar{\kappa}_{\mathrm{H}} = \kappa_0\gamma/(\gamma-1)$. These bounds are reported in Eq. (8) of the main text, and depicted in main text Figure 5.

3.3.2 Sharper bounds for $g > 1/2$

In the case $g > 1/2$, we can use the bounds in Supplementary Equation 58. From Supplementary Equations 77 and 78, we have

$$\begin{aligned}
\bar{\kappa}_{\mathrm{low}} &= \frac{\gamma \kappa_0}{2(\gamma-1)\big(1-2^{-\gamma/(\gamma-1)}\big)}, \\
\bar{\kappa}_{\mathrm{high}} &= \frac{\gamma \kappa_0}{2(\gamma-1)2^{-\gamma/(\gamma-1)}}.
\end{aligned}$$

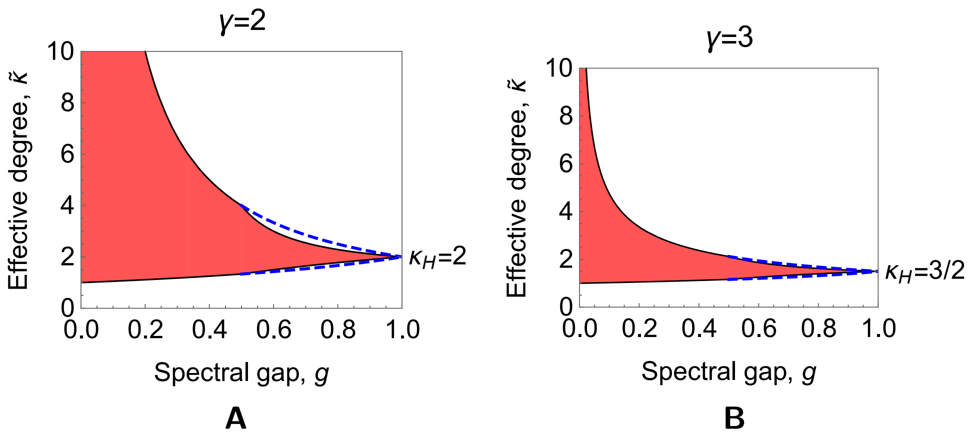

Supplementary Figure 1: Upper and lower bounds on the effective degree for graphs with power-law distribution of Simpson degrees: $f(\kappa) \propto \kappa^{-\gamma}$, for $\kappa \in [1, \infty)$. For $g < 1/2$ we use the bounds in Supplementary Equation 79, while for $g > 1/2$ we use the sharper bounds in Supplementary Equation 80, showing also the bounds in Supplementary Equation 79 for comparison.

This leads to

$$\left(\frac{\gamma}{\gamma-1}\right)\frac{g\kappa_0}{2g-1+(1-g)2^{-1/(\gamma-1)}} \leq \left(\frac{b}{c}\right)^* \leq \left(\frac{\gamma}{\gamma-1}\right)\frac{g\kappa_0}{1-(1-g)2^{-1/(\gamma-1)}}. \tag{80}$$

Supplementary Figure 1 illustrates these bounds for $\gamma = 2$ and $\gamma = 3$, both with $\kappa_0 = 1$. In both cases, the improvement over the bounds in Supplementary Equation 79 is relatively small.

Supplementary Note 4: Diffusible public goods

Here we provide derivations for the model described in 'Diffusible public goods' under main text results.

We begin as before with an undirected, connected isothermal graph G. In a given state, the occupant of each vertex $i \in G$ can be a producer ($x_i = 1$) or a nonproducer ($x_i = 0$). Producers pay a cost C to produce a public good. This public good diffuses according to a random walk from the producer, so that the individual at the nth step of this random walk receives benefit b_n, for

all $n \geq 0$. The sequence $b_0, b_1, b_2, \ldots$ is constrained only by the requirement that the total benefit $B = \sum_{n=0}^{\infty} b_n$ be finite.

With this setup, the payoff to vertex i in state $\mathbf{x}$ is

$$f_i(\mathbf{x}) = -Cx_i + \sum_{n=0}^{\infty} b_n x_i^{(n)}. \tag{81}$$

The expected payoff at the end of an m-step random walk from i is

$$f_i^{(m)}(\mathbf{x}) = -Cx_i^{(m)} + \sum_{n=0}^{\infty} b_n x_i^{(n+m)}. \tag{82}$$

The fraction of the total benefit that vertex j receives from a producer at vertex i is

$$\phi_{ij} = \frac{1}{B} \sum_{n=0}^{\infty} p_{ij}^{(n)} b_n. \tag{83}$$

A key quantity will be the expected fraction of public good received at the end of a k-step random walk from an initial vertex chosen proportionally to remeeting time:

$$\phi^{(k)} = \sum_{i \in G} \frac{\tau_i}{N^2} \sum_{j \in G} p_{ij}^{(k)} \phi_{ij}. \tag{84}$$

From Supplementary Equations 5 and 6 (which apply to this model as well as to pairwise games) we find that the producer type is favored under weak selection if and only if

$$\sum_{i \in G} \left\langle x_i \left(f_i - f_i^{(m)} \right) \right\rangle > 0, \tag{85}$$

where $m = 1$ for Bd or Db updating, and $m = 2$ for dB or bD updating. Substituting the payoffs from Supplementary Equation 81 and simplifying, Supplementary Equation 85 becomes

$$-C \sum_{i \in G} \left\langle x_i \left(x_i - x_i^{(m)} \right) \right\rangle + \sum_{n=0}^{\infty} b_n \sum_{i \in G} \left\langle x_i \left(x_i^{(n)} - x_i^{(n+m)} \right) \right\rangle > 0. \tag{86}$$

Applying Supplementary Equation 18, this condition becomes

$$-C\tau^{(m)} + \sum_{n=0}^{\infty} b_n \left(\tau^{(n+m)} - \tau^{(n)} \right) > 0. \tag{87}$$

Let us evaluate the benefit term. Iterating Supplementary Equation 11, we find

$$\begin{aligned}\tau^{(n+m)} - \tau^{(n)} &= \frac{1}{N}\sum_{i\in G}\tau_i \sum_{\ell=n}^{n+m-1} p_{ii}^{(\ell)} - m \\ &= \frac{1}{N}\sum_{i\in G}\tau_i \sum_{\ell=n}^{n+m-1}\left(p_{ii}^{(\ell)} - \frac{1}{N}\right).\end{aligned}$$

Substituting into the second term on the left-hand side of Supplementary

Equation 87, we compute

$$
\begin{aligned}
\sum_{n=0}^{\infty} b_n \left(\tau^{(n+m)} - \tau^{(n)}\right) &= \frac{1}{N} \sum_{n=0}^{\infty} b_n \sum_{i \in G} \tau_i \sum_{\ell=n}^{n+m-1} \left(p_{ii}^{(\ell)} - \frac{1}{N} \right) \\
&= \frac{1}{N} \sum_{i \in G} \tau_i \sum_{n=0}^{\infty} b_n \sum_{\ell=n}^{n+m-1} \left(\sum_{j \in G} p_{ij}^{(n)} p_{ji}^{(\ell-n)} - \frac{1}{N} \right) \\
&= \frac{1}{N} \sum_{i \in G} \tau_i \sum_{n=0}^{\infty} b_n \sum_{k=0}^{m-1} \left(\sum_{j \in G} p_{ij}^{(n)} p_{ji}^{(k)} - \frac{1}{N} \right) \\
&= \frac{1}{N} \sum_{i \in G} \tau_i \sum_{n=0}^{\infty} b_n \sum_{k=0}^{m-1} \sum_{j \in G} p_{ij}^{(n)} \left(p_{ji}^{(k)} - \frac{1}{N} \right) \\
&= \frac{1}{N} \sum_{i \in G} \tau_i \sum_{j \in G} \sum_{n=0}^{\infty} p_{ij}^{(n)} b_n \sum_{k=0}^{m-1} \left(p_{ji}^{(k)} - \frac{1}{N} \right) \\
&= \frac{B}{N} \sum_{i \in G} \tau_i \sum_{j \in G} \phi_{ij} \sum_{k=0}^{m-1} \left(p_{ij}^{(k)} - \frac{1}{N} \right) \\
&= \frac{B}{N} \sum_{i \in G} \tau_i \sum_{k=0}^{m-1} \left(\sum_{j \in G} p_{ij}^{(k)} \phi_{ij} - \frac{1}{N} \right) \\
&= NB \sum_{k=0}^{m-1} \sum_{i \in G} \frac{\tau_i}{N^2} \left(\sum_{j \in G} p_{ij}^{(k)} \phi_{ij} - \frac{1}{N} \right) \\
&= NB \sum_{k=0}^{m-1} \left(\phi^{(k)} - \frac{1}{N} \right) \\
&= B \left(N \sum_{k=0}^{m-1} \phi^{(k)} - m \right). \qquad (88)
\end{aligned}
$$

In rearranging the above sums, we have used that $\sum_{j \in G} p_{ij}^{(n)} = \sum_{j \in G} \phi_{ij} = 1$ for all $i \in G$ and $n \geq 0$, that $\sum_{i \in G} \frac{\tau_i}{N^2} = 1$, and that $p_{ij}^{(k)} = p_{ji}^{(k)}$ for all $i, j \in G$ and $k \geq 0$.

Supplementary Equation 88 shows how the benefits of public goods sharing are partially cancelled by spatial competition, depending on the scale m at which this competition occurs ($m = 1$ for Bd or Db, $m = 2$ for dB or bD). Only benefits that accrue at scales $k = 0$ through $k = m - 1$ contribute to

the success of the producer type, as shown by the presence of the terms $\phi^{(k)}$ for $0 \leq k \leq m-1$, but for not $k \geq m$, in Supplementary Equation 88.

The cost term of Supplementary Equation 87 becomes

$$
\begin{aligned}
-C\tau^{(m)} &= -C\left(\frac{1}{N}\sum_{i\in G}\tau_i \sum_{k=0}^{m-1} p_{ii}^{(k)} - m\right) \\
&= -C\left(N\sum_{k=0}^{m-1} p^{(k)} - m\right). \qquad (89)
\end{aligned}
$$

Above, we have defined $p^{(k)} = \sum_{i\in G} \frac{\tau_i}{N^2} p_{ii}^{(k)}$ to be the probability that a random walk visits its initial vertex at the kth step, where the initial vertex is chosen proportionally to remeeting time. In particular, we have $p^{(0)} = 1$, $p^{(1)} = 0$, and $p^{(2)} = 1/\tilde{\kappa}$. Substituting the appropriate values of m in Supplementary Equations 87, 88, and 89, we obtain the conditions

$$-C(N-1) + B\left(N\phi^{(0)} - 1\right) > 0 \qquad \text{Bd or Db} \qquad (90)$$

$$-C(N-2) + B\left(N\left(\phi^{(0)} + \phi^{(1)}\right) - 2\right) > 0 \qquad \text{dB or bD.} \qquad (91)$$

Supplementary Note 5: Non-isothermalizable topologies

We say that a given graph topology is *isothermalizable* if there exists a set of edge weights $\{w_{ij}\}$ consistent with the topology (in the sense that only those edges present in the topology can have positive weight), such that the resulting weighted graph is isothermal and connected.

Not all graph topologies have this property. For example, if a graph topology contains a leaf (a vertex of topological degree one), then the edge connecting this vertex to its neighbor must have weight one; consequently, this neighbor cannot have any other neighbors of positive edge weight. Thus, any topology of size $N \geq 3$ that contains a leaf is non-isothermalizable. As another example, a complete bipartite graph topology is isothermalizable only if the two partite sets have the same size.

For the preferential attachment model, non-isothermalizable topologies occurred in significant numbers occurred when the linking number m is small and the shift parameter a is close to 1; see Supplementary Figure 2. We removed such graphs from the ensemble.

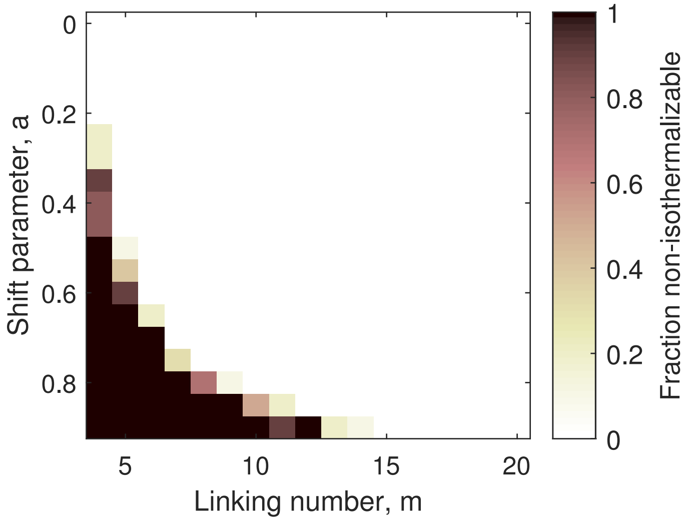

Supplementary Figure 2: Fraction of topologies generated by the shifted-linear preferential attachment model for which no isothermal weighting exists. Such non-isothermalizable topologies arise when the linking number m is small, and the shift parameter a is close to 1.

Supplementary References

[1] Benjamin Allen, Gabor Lippner, Yu-Ting Chen, Babak Fotouhi, Naghmeh Momeni, Shing-Tung Yau, and Martin A Nowak. Evolutionary dynamics on any population structure. *Nature*, 544(7649):227, 2017.

[2] Benjamin Allen and Alex McAvoy. A mathematical formalism for natural selection with arbitrary spatial and genetic structure. *Journal of Mathematical Biology*, 78(4):1147–1210, 2019.

[3] Benjamin Allen and Corina E Tarnita. Measures of success in a class of evolutionary models with fixed population size and structure. *Journal of Mathematical Biology*, 68(1-2):109–143, 2014.

[4] Richard A Holley and Thomas M Liggett. Ergodic theorems for weakly interacting infinite systems and the voter model. *The Annals of Probability*, 3(4):643–663, 1975.

[5] Thomas M Liggett. *Interacting Particle Systems*. Springer Science & Business Media, 2006.

[6] Corina E. Tarnita, Hisashi Ohtsuki, Tibor Antal, Feng Fu, and Martin A. Nowak. Strategy selection in structured populations. *Journal of Theoretical Biology*, 259(3):570 – 581, 2009.

[7] David Aldous and James Allen Fill. Reversible Markov Chains and Random Walks on Graphs. Unfinished monograph, recompiled 2014, available at `http://www.stat.berkeley.edu/aldous/RWG/book.html`, 2002.

[8] Joseph L Doob. *Stochastic Processes*. Wiley, New York, 1953.
