# Supplementary material for: Evolutionary games on isothermal graphs
Source: Nat Commun. 2019 Nov 8;10:5107. doi: 10.1038/s41467-019-13006-7 (PMC6841731; doi:10.1038/s41467-019-13006-7)
Supplement: Supplementary file 3 — Reporting Summary [file 41467_2019_13006_MOESM3_ESM.pdf]

nature research

Corresponding author(s): Benjamin Allen

Last updated by author(s): Sep 27, 2019

Reporting Summary

Nature Research wishes to improve the reproducibility of the work that we publish. This form provides structure for consistency and transparency in reporting. For further information on Nature Research policies, see Authors & Referees and the Editorial Policy Checklist.

Statistics

For all statistical analyses, confirm that the following items are present in the figure legend, table legend, main text, or Methods section.

n/a	Confirmed	
☐	☒	The exact sample size (n) for each experimental group/condition, given as a discrete number and unit of measurement
☒	☐	A statement on whether measurements were taken from distinct samples or whether the same sample was measured repeatedly
☒	☐	The statistical test(s) used AND whether they are one- or two-sided<br>*Only common tests should be described solely by name; describe more complex techniques in the Methods section.*
☒	☐	A description of all covariates tested
☒	☐	A description of any assumptions or corrections, such as tests of normality and adjustment for multiple comparisons
☒	☐	A full description of the statistical parameters including central tendency (e.g. means) or other basic estimates (e.g. regression coefficient) AND variation (e.g. standard deviation) or associated estimates of uncertainty (e.g. confidence intervals)
☒	☐	For null hypothesis testing, the test statistic (e.g. F, t, r) with confidence intervals, effect sizes, degrees of freedom and P value noted<br>*Give P values as exact values whenever suitable.*
☒	☐	For Bayesian analysis, information on the choice of priors and Markov chain Monte Carlo settings
☒	☐	For hierarchical and complex designs, identification of the appropriate level for tests and full reporting of outcomes
☒	☐	Estimates of effect sizes (e.g. Cohen's d, Pearson's r), indicating how they were calculated

Our web collection on statistics for biologists contains articles on many of the points above.

Software and code

Policy information about availability of computer code

Data collection	Data was generated using MATLAB version R2018B. We wrote custom MATLAB code, which we make available in Zenodo with the identifier http://dx.doi.org/10.5281/zenodo.3462156.
Data analysis	Data was analyzed using MATLAB version R2018B. We wrote custom MATLAB code, which we make available in Zenodo with the identifier http://dx.doi.org/10.5281/zenodo.3462156.

For manuscripts utilizing custom algorithms or software that are central to the research but not yet described in published literature, software must be made available to editors/reviewers. We strongly encourage code deposition in a community repository (e.g. GitHub). See the Nature Research guidelines for submitting code & software for further information.

Data

Policy information about availability of data

All manuscripts must include a data availability statement. This statement should provide the following information, where applicable:

- Accession codes, unique identifiers, or web links for publicly available datasets
- A list of figures that have associated raw data
- A description of any restrictions on data availability

All data generated or analysed during this study are included in this article and its supplementary information files.

Field-specific reporting

Please select the one below that is the best fit for your research. If you are not sure, read the appropriate sections before making your selection.

☐ Life sciences ☐ Behavioural & social sciences ☒ Ecological, evolutionary & environmental sciences

October 2018

Ecological, evolutionary & environmental sciences study design

All studies must disclose on these points even when the disclosure is negative.

Study description
We studied the conditions for success in evolutionary games on isothermal graphs. The vast majority of our results are based on mathematical derivations and proofs, which are provided in the Supplementary Information. Additionally, there were some numerical experiments on random graphs, which were implemented using MATLAB.

Research sample
Numerical data were generated using MATLAB code, which is made available in Zenodo with the identifier http://dx.doi.org/10.5281/zenodo.3462156.

Sampling strategy
For the spatial model (Fig. 3AC), the population size is $N=200$, the decay parameter is $\beta=2^{\ell/2}$ for $\ell=6,7,\ldots,14$, and the number of pairing rounds is $100,200,\ldots, 800$. Ten random isothermal graphs were generated for each parameter combination. The clusters of points in Figure 3C correspond to different values of β.

The shifted-linear preferential attachment model (Fig. 3BD) is defined as follows: Starting from a complete graph of size $m+1$, new vertices were added one at a time, each linking to m existing vertices, chosen with probability proportional to $k - am$, where k is vertex degree and a is a shift parameter. The process was iterated until the graph reached size $N=400$. We used linking numbers $m = 4,5,\dots, 20$, and shift parameter a varying from 0 to 0.9 in increments of 0.05. For each combination of a and m, we generated ten graph topologies. For each topology generated this way, an isothermal weighting was obtained by minimizing $\sum_{i,j} w_{ij}^2$ under the constraint $\sum_j w_{ij}=1$ for all i, using a numerical quadratic programming algorithm. This sum-of-squares minimization was chosen in order produce a unique set of edge weights that are relatively even---and therefore have relatively large Simpson degrees---given the constraints imposed by the topology and isothermality. Graph topologies that could not be made isothermal were removed from the ensemble; such graphs arose for small m and a close to 1.

Data collection
Data were generated using the MATLAB code, as written and exectued by Gabor Lippner

Timing and spatial scale
Not applicable

Data exclusions
For the preferential attachment model, graph topologies that could not be made isothermal were removed from the ensemble; such graphs arose for small m and a close to 1. We describe this exclusion in detail in Supplementary Note 5.

Reproducibility
All numerical data can be reproduced using the provided MATLAB code

Randomization
Not applicable

Blinding
No blinding was needed for the computer-generated data

Did the study involve field work? ☐ Yes ☒ No

Reporting for specific materials, systems and methods

We require information from authors about some types of materials, experimental systems and methods used in many studies. Here, indicate whether each material, system or method listed is relevant to your study. If you are not sure if a list item applies to your research, read the appropriate section before selecting a response.

Materials & experimental systems

n/a	Involved in the study
☒	☐ Antibodies
☒	☐ Eukaryotic cell lines
☒	☐ Palaeontology
☒	☐ Animals and other organisms
☒	☐ Human research participants
☒	☐ Clinical data

Methods

n/a	Involved in the study
☒	☐ ChIP-seq
☒	☐ Flow cytometry
☒	☐ MRI-based neuroimaging
